# How are caregivers involved in treatment decision making for older people with dementia and a new diagnosis of cancer?

**DOI:** 10.1002/pon.5070

**Published:** 2019-04-24

**Authors:** Charlene Martin, Anne Shrestha, Maria Burton, Karen Collins, Lynda Wyld

**Affiliations:** ^1^ Department of Oncology and Metabolism The University of Sheffield Sheffield; ^2^ Department of Health and Social Care Research Sheffield Hallam University Sheffield

**Keywords:** caregivers, decision making, dementia, neoplasms, proxy

## Abstract

**Objective:**

To explore how caregivers are involved in making treatment decisions for older people living with dementia and a new diagnosis of cancer.

**Method:**

A systematic review of PubMed, CINAHL, PsycINFO, Web of Science, and Scopus databases was conducted. Studies recruiting formal or informal caregivers for older people with dementia and a diagnosis of cancer were considered for inclusion.

**Results:**

Of 1761 articles screened, 36 full texts were assessed for eligibility, and six were included in the review. This review has identified that health care professionals (HCPs) are often unaware of the coexistence or severity of dementia in cancer patients, and therefore fail to properly address care needs as a result. While caregivers are relied on to help make decisions, they have unmet information needs and feel excluded from decision‐making.

**Conclusion:**

Treatment decision making in the context of older adults with dementia and a new diagnosis of cancer needs further research. This will help HCPs to understand their needs and improve the experience of decision making for both caregivers and the people that they care for.

## 1 BACKGROUND

Over the past 25 years, there has been a substantial growth in the older adult population, who now represent the fastest growing demographic in the United Kingdom.^1^ Older adults are now living much longer with advanced stages of age‐related comorbidities, such as cognitive impairment, and have an increased vulnerability to age‐related disease and cancers. The global incidence of dementia is approximately 10 million new cases each year,^2^ with around 850 000 people diagnosed with some form of dementia in the United Kingdom.^3^ The demographic outlook suggests that by 2021 an estimated 1 000 000 people will be living with some form of dementia. Advancing age is also a significant risk factor for cancer, with over half of all cancers being diagnosed in the over 70 age group each year in the United Kingdom.^4^

Dementia is a disease characterized by a progressive set of conditions that include loss of judgment, reasoning ability, and memory; all of which will impair capacity to make informed decisions.^5^ The scope of dementia impacts so much more than just memory; it can impair language, perception, and the ability to undertake daily tasks without additional care and assistance. Together, these changes over time can place a profound burden on their family and caregivers, especially in the later stages of dementia, which will increase the need for care services, psychosocial support, and assistance with treatment decision making.^6^ Dementia severity and the trajectory of functional decline vary from person‐to‐person, meaning that older adults who live with this condition are highly heterogeneous. Some people with memory problems may be living without a formal diagnosis or may avoid visiting their GP through fear and stigma of the disease.^7^ While some older adults in the early stages of the disease may have mild impairment and can still make informed decisions, others in the severe or later stages of dementia will rely on the input of others, such as their family and caregivers.^8^

Caregivers can either be formally or informally appointed to help make decisions on behalf of a person who lacks the capacity to make their own decisions.^9^ Some caregivers may be legally appointed to this role while the individual still has capacity, while others may take on this role suddenly or gradually over time. Under current U.K. law, caregivers must have lasting power of attorney (LPA) for health and welfare in order to make treatment‐related decisions on behalf of another person. The role of the caregiver in this scenario is to elicit the preferences of the person living with dementia and navigate, which treatments are in their best interests.^10^ In the literature, this is often referred to as proxy or surrogate decision making.

As the aging process varies, some older adults may be considered fitter than others and able to withstand different levels of treatment. Treatments for new cancer diagnoses should therefore be tailored and take into account any existing comorbidities and treatment regimes.^6^ For older adults with normal cognition, following routine clinical investigations and diagnosis, there will be a discussion about the pros and cons of treatment options together with the treating clinician before making an informed decision. Family, caregivers, and friends may be involved in the consultation and offer their support, but ultimately, the patient will make this decision and give their consent to treatment.

For people living with dementia, the process of diagnosis may differ significantly to those with no cognitive impairment; screening opportunities may be limited, and undergoing diagnostic investigations may present a burden to the person living with dementia and their caregiver.^11^ Clinical investigations such as scans and biopsies may also be more challenging. People with dementia may also lack the capacity to understand the treatment options available, and information may need to be adapted or presented in a manner adapted to their cognitive capacity. The language deficits frequently associated with severe dementia may mean that some people living with the disease are not able to communicate their decision and give verbal consent to treatment. Caregiver involvement may therefore be needed to interpret the patient's wishes and help guide the consultant towards a treatment plan that takes into account the wishes of the patient and is in their best interests.

In the United Kingdom, there are ethical frameworks that guide treatment decision making for both caregivers and clinicians,^12^ and there are legal guidelines that help individuals state their treatment preferences while they still have capacity to do so.^13^ The concepts of autonomy and informed decision making are paramount and should be upheld in cases where treatment preferences have been stated in an advance directive (AD) before the person lost capacity.^14^ An AD designates instructions for future medical treatment or for a decision maker with LPA to act on their behalf. Advance decisions are useful because they allow people to retain autonomy over their future treatment, particularly in cases where there is no opportunity to have a discussion about treatments. The reality is that not all people are able to state their preferences in advance of losing capacity and may not always have an AD in place.^15^ As a result of these issues, caregivers and clinicians may be presented with ambiguous circumstances where the preferences of the person they care for are unknown or have not been previously stated. In cases where it is not possible to determine which treatments the patient might decide for himself or herself, the principle of best interests should be used, and a best interest meeting may be held; involving clinicians, caregivers, individuals with LPA and sometimes even the person with dementia themselves.

Little is currently known about how caregivers are involved in making cancer treatment decisions for the older, cognitively impaired population. Previous reviews have struggled to identify many studies that have directly explored the experiences of people with dementia and their caregivers in this context.^16^ A recent review by Hopkinson and colleagues, which sought to find out about the experiences of people with cancer and dementia, found that people with dementia were more likely to have a delayed diagnosis and receive fewer treatments compared with cancer patient who did not have dementia.^17^

The aim of this review was to address a gap in knowledge by exploring how caregivers are involved in making cancer treatment decisions for older people with dementia who receive a new diagnosis of cancer.

## 2 METHODS

### 2.1 Search question

How are caregivers involved in making treatment decisions for older people with dementia and a new diagnosis of cancer?

### 2.2 Search strategy

A comprehensive search of the literature was conducted in accordance with PRISMA guidelines^18^ between June and September 2018 and revised again in January 2019. The following databases were searched: CINAHL, PubMed (via MEDLINE), PsycINFO, Scopus, and Web of Science. Hand‐searching reference lists and the gray literature also obtained references. The search was limited to the English language. Given the nature of the research question, an adapted “PCO” framework^19^ was used for this review. A broad range of key search terms and MeSH topics were used based around the topics of “decision making,” “caregivers,” “dementia,” and “cancer.” A combination of free text searches and MeSH terms were used to identify articles. An example of the search strategy for PubMed (via MEDLINE) is shown in Data S1.

### 2.3 Eligibility criteria

The search aimed to identify qualitative, quantitative, or mixed method studies that recruited caregivers (both informal and formal) for people living with dementia. Studies were included if they made reference to cancer treatment decision making for older people living with dementia. This included studies that observed treatment discussions in consultations and caregiver perspectives on hypothetical treatment scenarios. Reviews, letters, case studies, editorials, and conference abstracts were excluded. Studies were limited to those that focused on older adults (>60), as this age is widely accepted as a lower cut‐off for chronological older age.^20^

### 2.4 Quality appraisal

**Figure 1:**
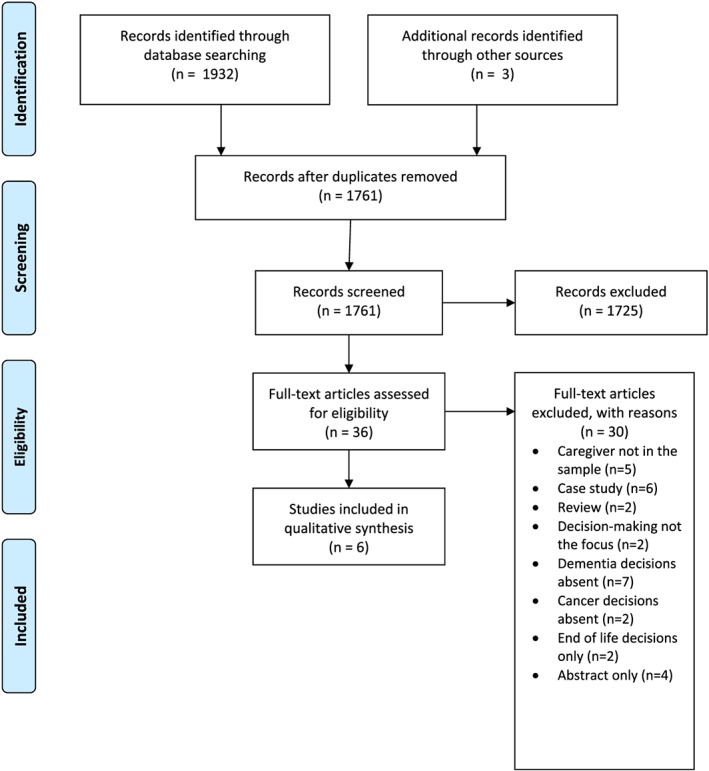
PRISMA flow diagram

Two reviewers (C.M. and A.S.) discussed and selected the articles included in this review. The rationale for including studies with either a mixed method, qualitative, or quantitative design was that this would allow a broad understanding of the research topic. Search results were imported to endnote for screening and full text retrieval. Studies were selected for this review using a two‐step process; articles were first screened by title and abstract to determine relevance to the review. The PRISMA search strategy^18^ was used to filter articles and remove any duplicates (Figure 1). Full text articles were then retrieved to assess relevance against the inclusion criteria and then independently reviewed.

The Mixed Method Appraisal Tool (MMAT)^21^, ^22^ quality checklist was used to appraise each study. The MMAT criteria include two screening questions and 19 items to appraise five types of study (qualitative, quantitative randomized controlled trials, quantitative nonrandomized, quantitative descriptive, and mixed methods). Quality assessment scores were calculated for each study using the MMAT score, ranging from one criteria met (25%) to all criteria met (100%). No study was excluded on the basis of quality assessment as the authors chose to include studies that represented the small amount of literature exploring decision making for people with dementia and cancer. However, for qualitative studies, the question “(1.4). Is appropriate consideration given to how findings relate to researchers' influence, e.g. through their interactions with participants?” was unclear or not always addressed.

### 2.5 Analysis

Thematic analysis was undertaken in accordance with the Framework approach.^23^, ^24^ This process involved coding the key findings across studies and then developing themes, which were then summarized within a framework matrix. Reviewing the matrix generated the final themes. The analysis was guided by an interpretivist approach.

## 3 RESULTS

The search produced a total of 1935 results. Of these, 174 duplicates were removed, and 1725 were excluded, as they did not meet the inclusion criteria. The remaining 36 articles were retrieved for full text review, and six of these were deemed suitable for inclusion. The term “caregiver” has been used throughout this paper to represent carers and informal caregivers. Health care provider (HCP) has been used as a comprehensive term for the treating clinician, consultant or oncology staff.

### 3.1 Study characteristics

Of the six studies included (Table 1), five were conducted in the United Kingdom and one in the United States. One study used a quantitative cross sectional design,^25^ and five studies used a qualitative design.^26^, ^27^, ^28^, ^29^, ^30^ Two studies explored treatment decision making in the context of hypothetical treatment scenarios,^25^, ^26^ three studies observed prospective treatment decision making in clinical scenarios,^27^, ^28^, ^29^ and one study interviewed patients and caregivers who were reflecting retrospectively on the cancer diagnosis and treatment decision making.^30^

**Table 1:** Studies included in the review

Author(s) and year of publication	Study Population and Setting	Objective(s)	Design	Method	Summary of Themes	MMAT score, %
Smyth (2009)	Family caregivers (n = 23) of women with dementia. Recruited from Alzheimer's Disease Research Center Registry, USA.	To explore breast screening and treatment decision‐making in older women with dementia.	Qualitative.	Semi‐structured telephone interviews. Thematic Analysis.	(1) Perceived importance of regular screening mammograms. (2) Perceived appropriateness of breast cancer treatment options.	75
Harrison Dening et al (2016)	Dyads of family caregivers and people with dementia (n = 60). Recruited from memory clinics in United Kingdom.	To explore choices and preferences of caregivers and people with dementia.	Quantitative. Cross sectional study.	Semi‐structured interviews. Descriptive statistics.	(1) Treatment choices and carer agreement in prediction. (2) Uncertainty. (3) Carer burden, distress, and quality of relationship.	75
Courtier et al (2016)	Caregivers and people with dementia. Thirty‐three consultations observed. 10 consultations recorded, 16 interviews (n = 6 patient‐caregiver dyads; n = 1 lone patient; n = 5 staff). Medical record review (n = 338). Recruited from four outpatient clinics in one UK cancer center.	To observe the management of patients with dementia, memory loss and cancer. Explore the needs and preferences of outpatient cancer services,	Qualitative Case Study Design.	Retrospective case note review; observation; interviews; recoded consultations. Framework Analysis.	(1). Memory and the cancer consultation. (2) Staff attitudes. (3) Management Approach. (4) Carer role.	75
McWilliams et al (2018)	Informal caregivers (n = 9); people with dementia‐cancer (n = 10), and oncology HCPs (n = 12). Recruited from a regional NW England Cancer Centre, UK.	To explore the information needs and experiences of caregivers, patients with dementia‐cancer and oncology HCPs.	Qualitative. Cross Sectional Design.	Semi‐structured face‐to‐face interviews. Thematic analysis.	(1) Leading up to the cancer consultation. (2) Communicating clinically relevant information. (3) Adjustments to cancer care. (4) After cancer treatment finishes.	100
Witham et al (2018)	Informal family caregivers (n = 7). Recruited from a psycho‐oncology unit at a regional cancer center, UK.	To explore the experiences of caregivers of relatives with cancer and dementia.	Qualitative. Narrative Approach.	Semi‐structured face‐to‐face interviews using interview guide. Analytical Framework.	(1) Communication with Health Care Professionals: Maintaining carer identity. (2) Decision making and maintaining personhood. (3) Negotiating cancer care.	100
McWilliams et al (2018)	Family caregivers (n = 9); people with dementia‐cancer (n = 10). Recruited from a regional NW England Cancer Centre, UK.	To explore the decision‐making and treatment options for people with dementia‐cancer, and their family caregivers.	Qualitative. Exploratory.	Semi‐structured face‐to‐face interviews. Thematic approach.	(1) Reaching a diagnosis of cancer; (2) Adjusting to the cancer diagnosis when living with dementia; (3) Weighing up the cancer treatment options; (4) Undergoing cancer treatment.	100

Abbreviation: HCP, health care professional; MMAT, Mixed Method Appraisal Tool.

All six studies used semi‐structured interviews (face‐to‐face or telephone) with informal/familial caregivers to collect primary data. Five qualitative studies used a framework, thematic, or narrative analysis,^26^, ^27^, ^28^, ^29^, ^30^ and one study used descriptive statistics.^25^ Two studies observed or interviewed HCPs in addition to caregivers,^27^, ^28^ and four studies also included the views of people with dementia.^25^, ^27^, ^28^, ^30^

Four studies specified a clinical diagnosis of dementia for the patient being cared for in their inclusion criteria,^25^, ^28^, ^29^, ^30^ and one study included caregivers for patients with a memory problem, as judged by the HCP and patient.^27^ Five studies recruited participants from either cancer clinics^27^, ^28^, ^29^, ^30^ or memory clinics,^25^ and one study recruited caregivers from a dementia registry.^26^

Three studies reported their sampling method as purposive,^28^, ^29^, ^30^ while others were unclear.^25^, ^26^, ^27^ The sample sizes of interviewees reported in the qualitative studies ranged from six to 60 patient‐caregiver dyads,^25^, ^27^, ^30^ and seven to 23 caregivers interviewed individually.^26^, ^28^, ^29^ Two studies interviewed patients with dementia individually in their case sample.^28^, ^30^

Thematic analysis using established theorists such as Wolcott framework and Braun and Clarke thematic analysis were used in three qualitative studies,^27^, ^28^, ^30^ and Riessman narrative approach was used in one study.^29^ One study measured responses using caregiver specific questionnaires, such as the Quality of Carer Patient Relationship questionnaire (QCPR).^25^

### 3.2 Findings

Because of the study design of selected papers, a meta‐analysis was not possible. Data have been categorized into three themes that interplay with the caregiver experience of making treatment decisions: (a) HCP dementia awareness and knowledge in the clinical consultation; (b) treatment decision‐making discussions, information, and communication needs; (c) the caregiver role and the caregiver‐patient relationship.

#### 3.2.1 HCP dementia awareness and knowledge in the clinical consultation

Following screening, clinical investigation, and diagnosis, the initial cancer consultation was often the first point in the cancer treatment pathway where HCPs met with patients and their caregivers to discuss treatment options. Four studies explored the experiences of caregivers and HCPs in the cancer setting through observation of consultations^27^ and semi‐structured interviews.^27^, ^28^, ^29^ Caregivers in one study reflected back on the consultation where the person living with dementia received their cancer diagnosis.^30^

Having access to detailed patient information, such as past medical history, comorbidities, and cognition level enabled HCP's to plan sufficient time for discussion in the consultation.^28^ McWilliams and colleagues noted that cognition status was not always known to the HCP in advance of the consultation,^28^ and dementia was also infrequently documented in the patient's referral information or medical records in the study led by Courtier and colleagues.^27^ In both studies, the identification of memory problems was often reliant on caregiver disclosures.^27^, ^28^ As a result of this unawareness, one caregiver described a scenario where the HCP failed to acknowledge his wife's distress when undergoing a clinical investigation, alongside failing to fully explain what the procedure entailed and what was expected.^30^

In most studies, the caregiver accompanied the patient in the consultation where treatment options were discussed. In the study led by Witham and colleagues, one caregiver described a series of scenarios that led to missed appointments where the patient attended their appointment unassisted; this was due to unclear signage in the clinic and an absence of staff to guide the patient once in the hospital.^29^ The cognitively impaired patient also had a coexisting hearing problem and was unable to hear his name being called. Another caregiver in this study highlighted the logistics of transporting people with dementia who live on their own in the community to their appointments. A scenario was recalled where the patient's erratic sleep pattern was incompatible with the arranged transport pick‐up time. This meant that without prompts, the patient would miss their transport to the appointment.^29^

In the context of consultation discussions, caregivers felt that decisions had been made by HCP's prior to the initial consultation^28^ and relayed feeling excluded from decisions.^29^ Caregivers in McWilliams and colleagues' study highlighted the uncertainties around taking consent from people with dementia to undergo clinical investigations and the level of responsibility expected from caregivers.^30^ Caregivers also highlighted the need for additional appointments, where treatment plans could be discussed further, independently from the patient.^29^

Caregivers in the study led by Courtier and colleagues noted that the cancer consultation had a tendency to focus primarily on cancer treatments, rather than cognition‐related problems.^27^ This subsequently led to memory problems remaining undiscovered. In two studies, it was noted that patients would often underplay memory problems^27^ and dispute their inability to cope with treatment as a result of their impairment**.** 29 Cancer diagnostic investigations were often delayed due to the combination of limited HCP awareness of memory problems and a failure to detect the signs associated with dementia.^28^ The lack of timely organization of support for people with memory problems was therefore an issue.^27^, ^28^

Two studies highlighted that dementia awareness training for cancer clinicians was needed.^27^, ^28^ The reasons for this included a lack of awareness of the impact that dementia may have on cancer screening^28^ and the potential for interaction between the patients' dementia symptoms and cancer treatment.^27^ Being unaware of the patients' ability to give informed consent may result in HCPs taking the refusal of treatment at “face value,” as noted by a caregiver in the study by Witham et al.^29^ Witham and colleagues describe one situation where a patient failed to complete their radiotherapy treatment due to refusing to attend appointments. This scenario was a result of the HCP failing to acknowledge that the person with dementia lacked the capacity to make informed decisions. In examples where HCPs were made aware of cognition problems in the patient, there was an uncertainty on how to best support them.^27^

In Smyth's^26^ study of breast screening and treatment preferences, caregiver decisions were found to be influenced by HCPs in regards to continued breast imaging; with a tendency to continue screening based on the clinician's recommendations. Witham and colleagues also noted the dominance of the HCPs' knowledge in the consultation, through a scenario where a caregiver relayed feeling that their judgment of the patient's progress and response to treatment was challenged.^29^

The need to involve dementia‐specific support at the outset was emphasized by caregivers, with one study highlighting the example of a designated dementia nurse and biographical tool, which was used in clinic to enhance support for patients with dementia.^27^ Two studies highlighted the need for HCP familiarity and how this was accomplished by using a designated HCP to coordinate care.^28^, ^29^ This avoided the need for repetitive recall of the patients' medical history and unnecessary frustration and anxiety for the patient.^29^ McWilliams and colleagues reported a positive scenario where the caregiver found it helpful to have the HCP repeat the information to her husband and pay more attention to the pacing of the consultation, which led to a positive experience for both the caregiver and the person with dementia.^30^

#### 3.2.2 Treatment decision making discussions, information, and communication needs

Weighing up the pros and cons of treatment options for a person living with dementia may not only involve the caregiver; the HCP and patients themselves may also be involved in making these decisions. Five studies reported on the direct influence of dementia on cancer treatment discussions.^26^, ^27^, ^28^, ^29^, ^30^

For people with dementia or memory issues, extra time may be needed to communicate information about their cancer diagnosis and treatment options. One study highlighted a scenario where a caregiver relayed his mother's lack of awareness of her diagnosis due to her dementia and relayed a scenario of conveying the diagnosis to his mother using a “creative strategy” and metaphors.^30^ Caregivers in Witham and colleagues' study also described the need for “complex communication strategies.”^29^ Examples of this in other studies included taking more time to discuss options,^30^ “slowing down” information delivery and using a change in language to communicate complex treatment information.^28^

In situations where patients lacked capacity, the caregiver gathered treatment information and negotiated on behalf of the patient,^27^, ^29^ acting “as a reliable messenger” or “relayer of information” between the HCP and the patient.^28^, ^30^ In another study, one caregiver reflected on this role in assisting the HCP during his wife's cancer investigations; describing a scenario where he would stay in the room to reassure his wife and would break down complicated instructions from the HCP to his spouse.^30^

In respect to treatment decisions, caregivers in Smyth's study expressed the view that side effects would have an influence on the pursuit of any hypothetical cancer treatments, with some only willing to opt for active treatments when the side effects were less severe.^26^ It was noted in another study, however, that comprehensive treatment information on the risks and side effects were not always fully explained to caregivers, and often misunderstood.^28^ In respect to the level of information received, some caregivers reported receiving enough verbal information, such as leaflets, but others described feeling they had to seek information for themselves postconsultation.^30^

Smyth's study of current practices in breast cancer treatment found that dementia severity had an impact on the decision making of caregivers towards screening and hypothetical treatment scenarios.^26^ For caregivers of women with severe dementia, only comfort care was suggested, while in women with mild‐moderate dementia, caregivers were more likely to choose typically “aggressive” treatments. It was also noted that caregiver treatment decisions, while hypothetical, did not always take into consideration the patient's comorbidities and life expectancy.^26^

Courtier and colleagues highlighted that people with dementia are likely to receive less treatment than patients without dementia.^27^ Reasons for this include the implications of dementia on life expectancy and the inability to tolerate treatments with complex regimens and severe side effects.^27^ McWilliams and colleagues also noted that the combination of cognition and communication impairments had a direct influence on treatment options, particularly the potential for side effects,^28^ and the impact on quality of life for the person living with dementia was noted in another study.^30^

The impact of dementia on treatment was not always considered by HCPs, and there was little regard for how treatment pathways could be adapted to meet the patient's needs.^29^ In this context, Witham and associates posit that the adaption of treatment regimens is needed for this population.^29^ When discussing treatment options with the HCP, caregivers reported unmet information needs; whereby information was not always communicated in an appropriate format, nor adapted in a way that was specific to patients with a cancer‐dementia diagnosis.^28^

#### 3.2.3 The role of the caregiver and the caregiver‐patient relationship

The caregiver plays an important role in cancer treatment decision making, mainly by facilitating discussion around the treatment and care preferences of people who lack capacity. All six studies recruited caregivers.^25^, ^26^, ^27^, ^28^, ^29^, ^30^

In the study led by McWilliams and colleagues, caregivers played a role in both uncovering symptoms and seeking help for the person with dementia, describing these as “detective stories.”^30^ In other studies, family and informal caregivers were described as the key to a successful consultation^27^, ^28^ and best placed to represent the voice of the patient; particularly in scenarios where the caregiver knew the patient well.^27^ This point was also echoed in McWilliams and colleagues' study, whereby the researcher reflected on the significance of the “longitudinal and biographical” knowledge of the caregiver in research interviews.^30^ In some cases however, Witham and colleagues noted that patients were prone to downplaying the importance of the caregiver role and that this in turn meant that advocating on behalf of the patient could be challenging for their relatives.^29^

Caregivers are often relied upon to ensure that patients adhere safely to treatment and monitor any untoward side effects.^27^ New treatment regimens, additional appointments, and assisted home care needs may increase the demand on caregivers themselves, such as radiotherapy treatment, which may require repeated trips to hospital.^30^ These additional burdens on the caregiver were not always considered during treatment discussions,^28^ and HCPs were not always found to enquire about the needs of the caregiver.^27^ As a result, some caregivers reported feeling excluded from the patient's cancer journey.^29^ Caregivers in the same study felt that their role was often marginalized by the HCP; describing a scenario where their knowledge and judgment of the person with dementia was questioned by the HCP.^29^

It is posited that the caregiver‐patient relationship itself may have a direct impact on the outcome of treatment decisions. Courtier and colleagues noted that in scenarios where the caregiver did not know the patient personally, “memory loss acted as a barrier to a successful consultation.”^27^ The HCPs' reliance on informal and family caregivers was also highlighted by McWilliams and colleagues, who reported difficulties where patients with dementia had attended clinic with a caregiver who had limited knowledge and no relationship to the patient.^28^

People living with dementia often rely on caregivers to make decisions on their behalf. The HCPs in courtier's paper were happy to conduct consultations with the caregiver taking the lead decision‐making role; however, this could sometimes have the unintended effect of disguising memory difficulties experienced by the person with dementia, unless it was disclosed or made known to the clinician.^27^ However, Harrison Dening and colleagues reported that the dependency on caregivers to interpret patient decisions might be misplaced. In their study of hypothetical treatment scenarios, caregivers and patients did not always agree consistently on future treatment scenarios. Where asked about advanced cancer treatment scenarios, patients with dementia had a preference for antibiotic treatment (47%) over CPR (30%) and tube feeding (37%). Within dyads, there was a low level of agreement.^25^

## 4 DISCUSSION

The key findings from this review highlight the lack of dementia specific support at the start of the cancer journey. People with dementia require additional support, and time for discussion, when planning treatment and attending appointments. While caregivers are often relied upon for their biographical knowledge, their support and information needs are not always considered by HCPs. These findings show that there is a missed opportunity for allowing caregivers a more active role in consultations and treatment decision making for people with dementia and cancer.

The main aim of this review was to explore how caregivers are involved in making treatment decisions for older people living dementia who receive a new diagnosis of cancer. This aim, however, was only partially achieved. One reason for this was the limited scope of studies that have focused specifically on caregiving for this subpopulation. The intention was to review studies that explored treatment decision making in the context of early stage cancer, with a focus on life‐sustaining treatment, rather than end of life treatment decision making. However, very few studies could be found in the initial scoping stages of the review.

Although the search strategy focused on studies that recruited caregivers, one theme emerging from this review is the notion that discussions around memory, behavioral and psychological symptoms (BPSD) of dementia are absent from the cancer consultation. It is therefore unclear if these symptoms are taken into account when discussing the suitability of different treatments. The studies included in this review have highlighted some of the barriers to navigating health care appointments and treatment discussions. These issues are consistent with the wider literature, which highlights the complexities involved with HCP encounters for people with dementia.^31^

While some of the studies included in this review have explored the impact of dementia on treatment decision making, there has been insufficient focus on how caregivers make treatment decisions, the type of information that caregivers would prefer to receive, and how advance decisions are used in the decision‐making discussions. Only one study made reference to the theme of maintaining the “pre‐dementia” preferences of people with dementia in respect to breast screening; however, these preferences were not upheld as the severity of dementia increased.^26^

A significant finding from the literature was the lack of knowledge and dementia awareness among health care professionals. There may be other complex issues influencing the treatment decision that have not been fully addressed. This may include the age, frailty, mobility, and independence of the person with dementia.

### 4.1 Study limitations

The small number of studies in this review highlights the need for more research in to the cancer treatment decision‐making experiences of older people with dementia and their caregivers. One explanation for the lack of studies in this area may be that this population is difficult to access and obtain consent to participate in research studies. The settings for the studies included were cancer clinics,^27^, ^28^, ^29^, ^30^ dementia registries,^26^ and memory clinics,^25^ which are key settings for capturing patients with dementia or their caregivers. Despite this, recruitment was still challenging. Two studies reflected on the challenges in identifying participants, the consent process and small number of eligible participants.^25^, ^27^ Courtier and colleagues reflected on how their study sample was smaller than expected,^27^ hinting at underlying inequality in access to cancer services in those with a dementia diagnosis.

Of the studies included in this review, the level of cognitive impairment (mild/moderate/severe) and functioning of patients was not always clear, except in the two studies, which reported dementia subtypes.^29^, ^30^ It is therefore not possible to make generalizations regarding all older patients with dementia. It is not possible to make any assumptions about the experiences of caregivers for people with mild dementia verses severe dementia, and more research is needed to translate findings to a range of cancer populations.^27^

### 4.2 Clinical implications

The involvement of people living with dementia in research requires a high level of ethical scrutiny. In addition, there are strict safeguarding policies in place for any research involving participants with limited cognitive capacity. The small numbers of participants included in these papers hint at the complexities involved in recruitment and the additional support that caregivers and patients in this population may need to participate in research.

Low recruitment may also be linked to the sensitive nature of making decisions for another person, at what is undoubtedly a highly emotive time in their cancer journey. Receiving a cancer diagnosis can be psychologically stressful for both people with dementia and their caregivers. Therefore, deciding on the right time to approach caregivers might affect their willingness to take part in research. For this reason, many researchers may be cautious about causing distress, and caregivers may gate‐keep access to people with dementia.^32^

The themes identified in this review are consistent with the background context of dementia‐cancer research. This review has identified a clear need to increase specialist dementia support for both the patient and caregivers from the initial consultation and throughout the cancer care pathway. Ensuring that HCPs have appropriate training and can identify memory, behavioral and cognition problems will mean that any advice or treatment recommended is tailored appropriately to the patient. More specific information tailored towards caregivers and people living with dementia is also needed in order to optimize treatment decision making.

## 5 CONCLUSION

Cancer treatment decision making for older people with dementia remains a complex issue. With an ever‐increasing aged population, this research raises concerns about the management of people with cancer who lack mental capacity and the support needs for those who are directly involved in making difficult choices on behalf of the people they care for. Further exploration of caregiver experiences in this context is needed.

## CONFLICT OF INTEREST

The authors declare no conflict of interest.

## Supporting information

Data S1. APPENDIX 1: PUBMED SEARCH STRATEGY
