# Supplementary material for: How are caregivers involved in treatment decision making for older people with dementia and a new diagnosis of cancer?
Source: Psychooncology. 2019 Apr 24;28(6):1197–206. doi: 10.1002/pon.5070 (PMC6563536; doi:10.1002/pon.5070)
Supplement: Supplementary file 1 — Data S1. APPENDIX 1: PUBMED SEARCH STRATEGY [file PON-28-1197-s001.docx]

# 10. APPENDIX 1: PUBMED SEARCH STRATEGY

(("dementia"[MeSH Terms] AND "decision making"[MeSH Terms]) AND ("neoplasms"[MeSH Terms] OR cancer[Text Word])) AND ("caregivers"[MeSH Terms] OR carer[Text Word]).
